# Supplementary material for: Cyclophilin A Is Not Acetylated at Lysine-82 and Lysine-125 in Resting and Stimulated Platelets
Source: Int J Mol Sci. 2022 Jan 27;23(3):1469. doi: 10.3390/ijms23031469 (PMC8836233; doi:10.3390/ijms23031469)
Supplement: Supplementary file 1 [file ijms-23-01469-s001.zip › ijms-1545228-supplementary Table S1.pdf]

Supplementary Table 1: Clinical and preclinical CyPA concentrations

Clinical Parameters	nM (CyPA)	ng/mL (CyPA)	Ref
Healthy Control (n=50)	0.12	2.2	[16]
Healthy Control (n=20)	0.42	7.6	[17]
Unstable Angina (n=60)	0.67	12.1	[16]
Coronary Heart Disease (n=70)	0.77	13.8	[17]
Acute Myocardial Infarction (n=90)	0.77	13.9	[16]
Coronary Artery Disease (n=189)	2.7	<50	[18]
Myocardial Infarction 1 month (n=27)	3.13	56.3	[19]
Myocardial Infarction baseline t=0 (n=28)	3.65	65.7	[19]
**Preclinical Parameters**	**nM (CypA)**	**ng/mL (CyPA)**	
ICAM1, EC50	9.0	162	[20]
VCAM1, EC50	17.8	320.4	[20]
IkB, EC50	25.0	450	[20]
Platelet degranulation	100	1800	[21]
Thrombus formation)	200	3600	[21]
Prevents fibrin clot formation	555.56	10000	[22]
**Preclinical Parameters**	**nM (AcK-CyPA)**	**ng/mL (AcK-CyPA)**	
ICAM1, EC50	1.6	28.8	[20]
IκB, EC50	5.0	90	[20]
VCAM1, EC50	9.5	171	[20]
P-p65 (NF-κb), EC50	17.8	320.4	[20]
VSMC activation / proinflammatory	50	900	[23]

CyPA (Cyclophilin A), ICAM1 (Intercellular Adhesion Molecule 1 / CD54), IκB (IkappaB kinase), VCAM1 (vascular cell adhesion molecule 1 / CD106), VSMC (vascular smooth muscle cells) P-p65 (phosphorylated transcription factor RelA / nuclear factor NF-kappa-b NF-κb p65 subunit), AcK (acetylated lysine), EC50 (Effective Concentration 50%)

References

16. Yan, J.; Zang, X.; Chen, R.; Yuan, W.; Gong, J.; Wang, C.; Li, Y., The clinical implications of increased cyclophilin A levels in patients with acute coronary syndromes. Clinica chimica acta; international journal of clinical chemistry 2012, 413, (7-8), 691-5.
17. Al-Kraity, W. R. H.; Al-Dujaili, A. N. G., Assessment of Cyclophilin-A Level in women with heart disease after menopause. Res J Pharm Technol 2017, 10, (6), 1675-1678.
18. Satoh, K.; Fukumoto, Y.; Sugimura, K.; Miura, Y.; Aoki, T.; Nochioka, K.; Tatebe, S.; Miyamichi-Yamamoto, S.; Shimizu, T.; Osaki, S., Plasma cyclophilin A is a novel biomarker for coronary artery disease. Circ J 2012, CJ-12-0805.
19. Huang, C.-H.; Chang, C.-C.; Kuo, C.-L.; Huang, C.-S.; Lin, C.-S.; Liu, C.-S., Decrease in plasma cyclophilin A concentration at 1 month after myocardial infarction predicts better left ventricular performance and synchronicity at 6 months: a pilot study in patients with ST elevation myocardial infarction. Int J Biol Sci 2015, 11, (1), 38.
20. Xue, C.; Sowden, M.; Berk, B. C., Extracellular Cyclophilin A, Especially Acetylated, Causes Pulmonary Hypertension by Stimulating Endothelial Apoptosis, Redox Stress, and Inflammation. Arterioscler Thromb Vasc Biol 2017, 37, (6), 1138-1146.
21. Seizer, P.; Ungern-Sternberg, S. N. I. v.; Schönberger, T.; Borst, O.; Münzer, P.; Schmidt, E.-M.; Mack, A. F.; Heinzmann, D.; Chatterjee, M.; Langer, H.; Malešević, M.; Lang, F.; Gawaz, M.; Fischer, G.; May, A. E., Extracellular Cyclophilin A Activates Platelets Via EMMPRIN (CD147) and PI3K/Akt Signaling, Which Promotes Platelet Adhesion and Thrombus Formation In Vitro and In Vivo. Arteriosclerosis, Thrombosis, and Vascular Biology 2015, 35, (3), 655-663.
22. Morozov, Y. A.; Khromykh, L. M.; Dementieva, I. I.; Charnaia, M. A.; Kulikova, N. L.; Kazansky, D. B., Recombinant Human Cyclophilin A in vitro Inhibits the Formation of Fibrin Clot. Acta naturae 2012, 4, (2), 98-101.
23. Soe, N. N.; Sowden, M.; Baskaran, P.; Kim, Y.; Nigro, P.; Smolock, E. M.; Berk, B. C., Acetylation of cyclophilin A is required for its secretion and vascular cell activation. Cardiovasc Res 2013, 101, (3), 444-453.
