# Supplementary material for: Cyclophilin A Is Not Acetylated at Lysine-82 and Lysine-125 in Resting and Stimulated Platelets
Source: Int J Mol Sci. 2022 Jan 27;23(3):1469. doi: 10.3390/ijms23031469 (PMC8836233; doi:10.3390/ijms23031469)

Uncropped original blots

Figure 1

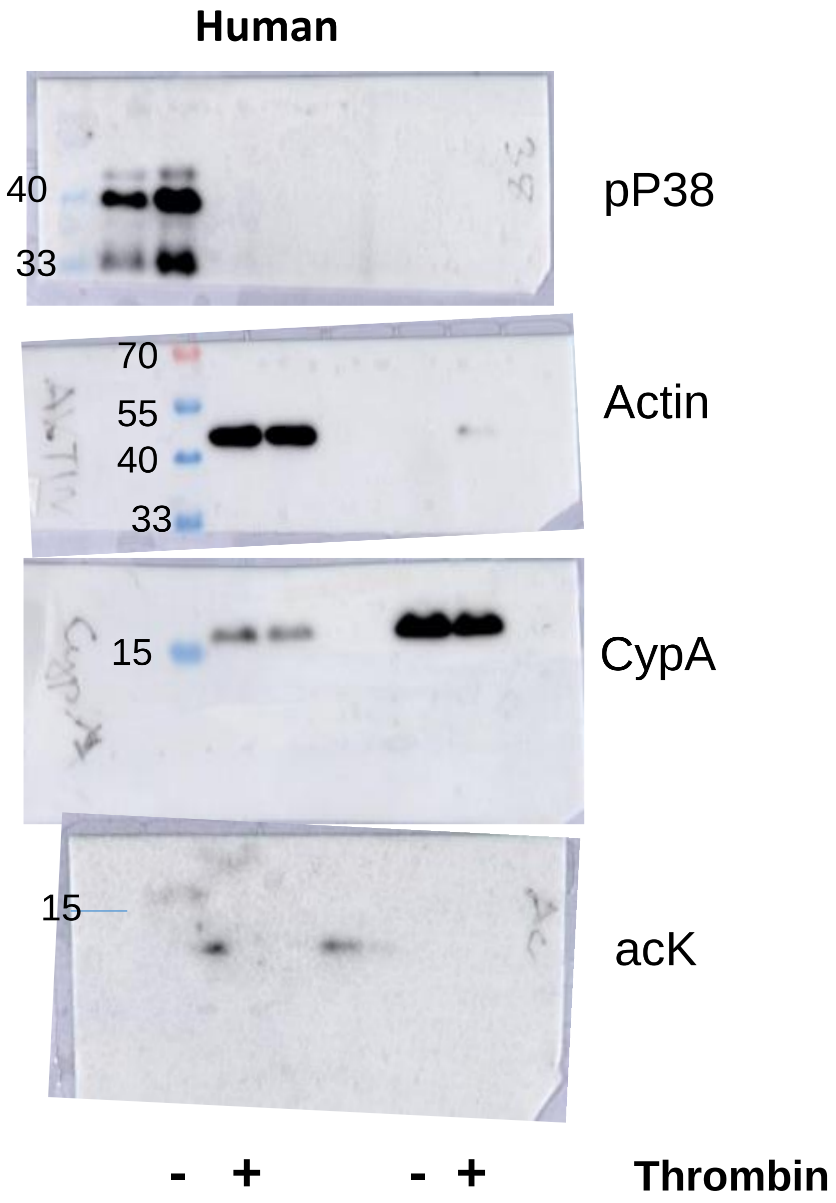

Mouse

Blots were generated in 2017 and at that time not saved as original tiff-files but just as cropped images.

We here include a second mouse blot showing similar results to underline results presented in Figure 1.

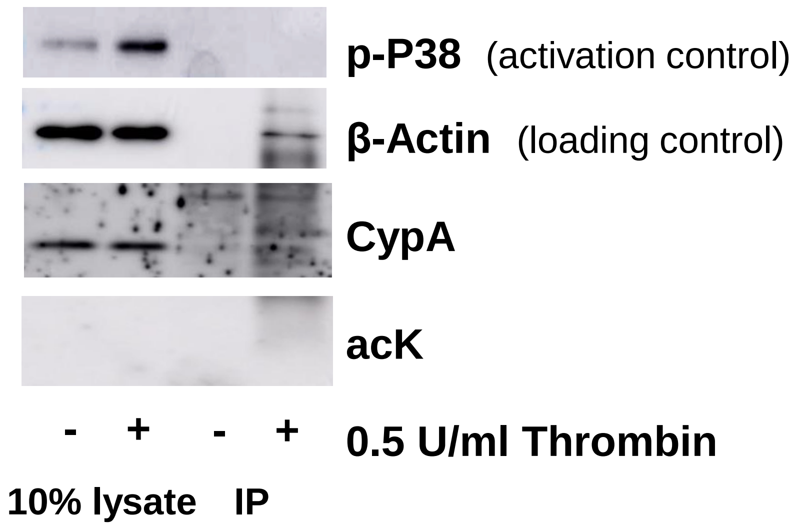

Uncropped original blots

Figure 2

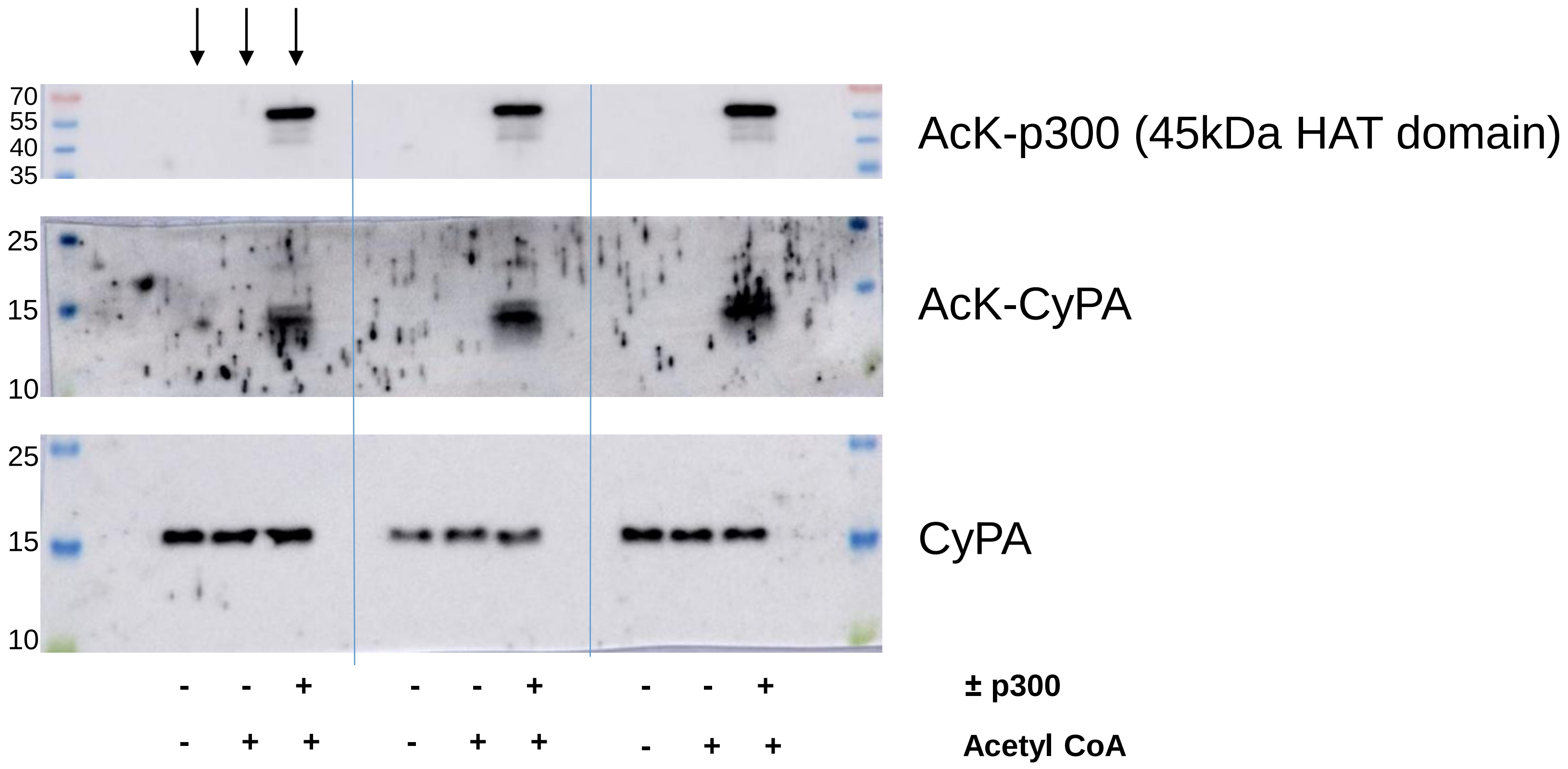

Uncropped original blots

Figure 3

Human platelet lysate **Mouse platelet lysate**

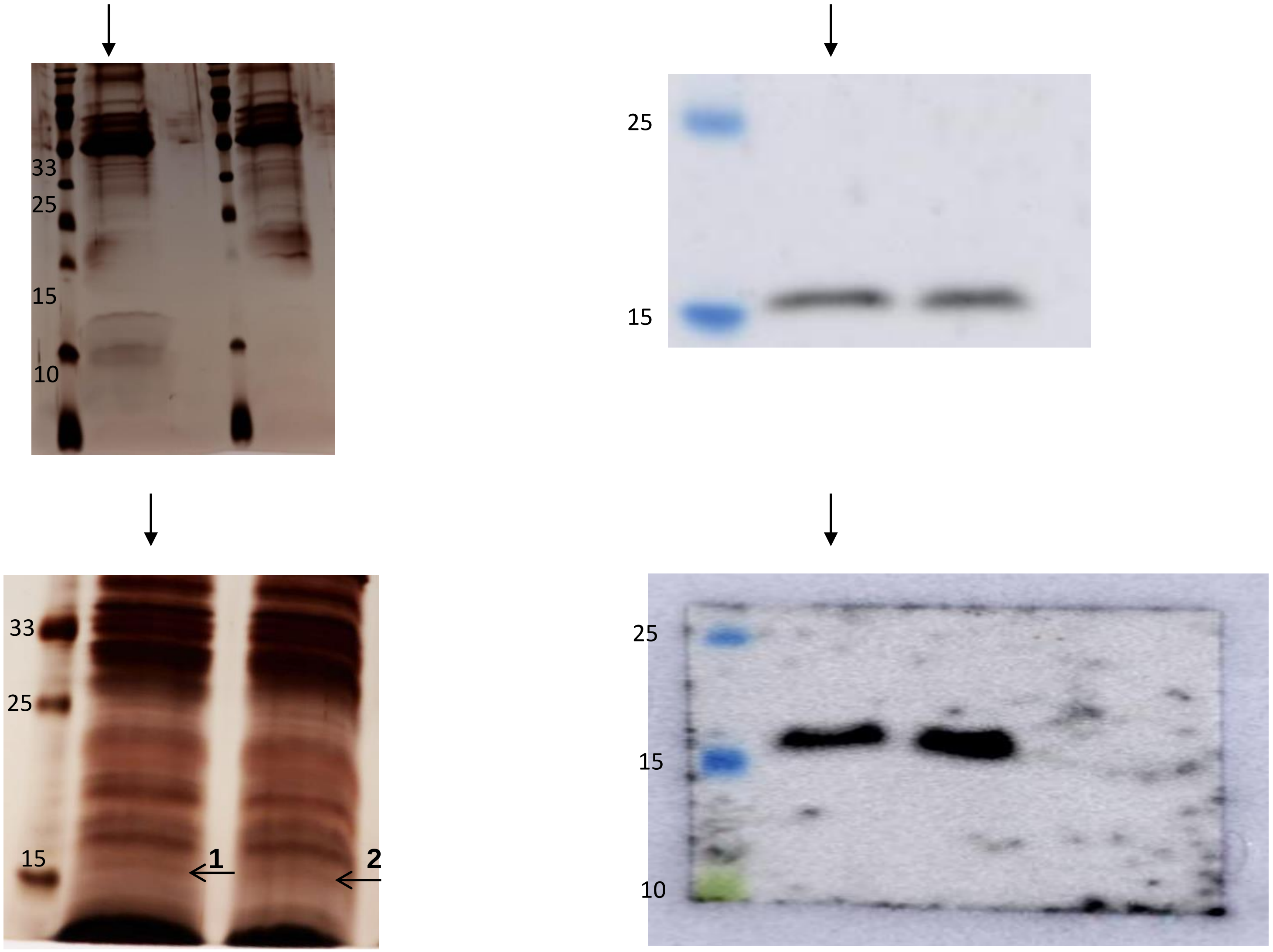

Uncropped original blots

Figure 4

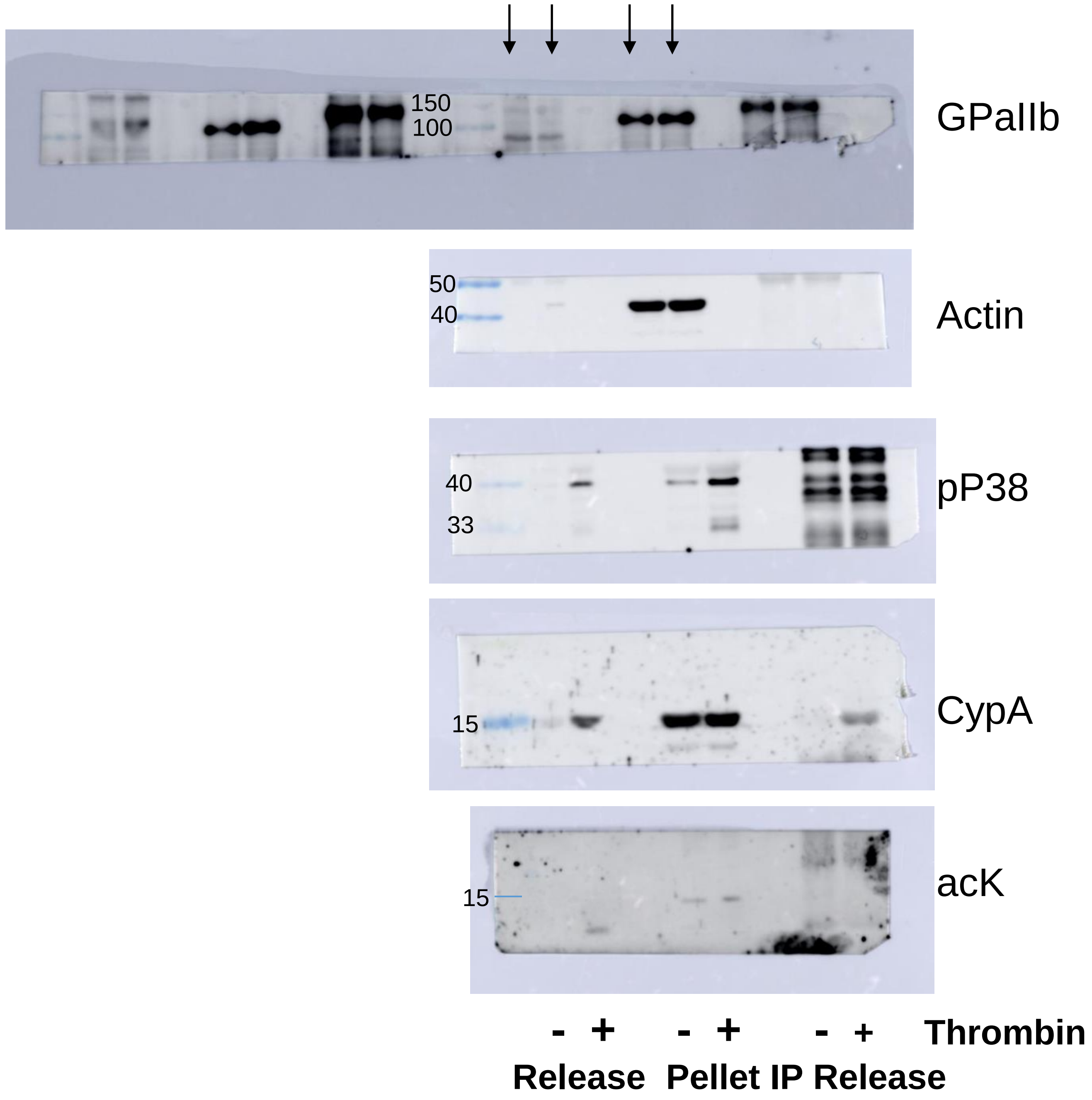

Uncropped original blots

Figure 5

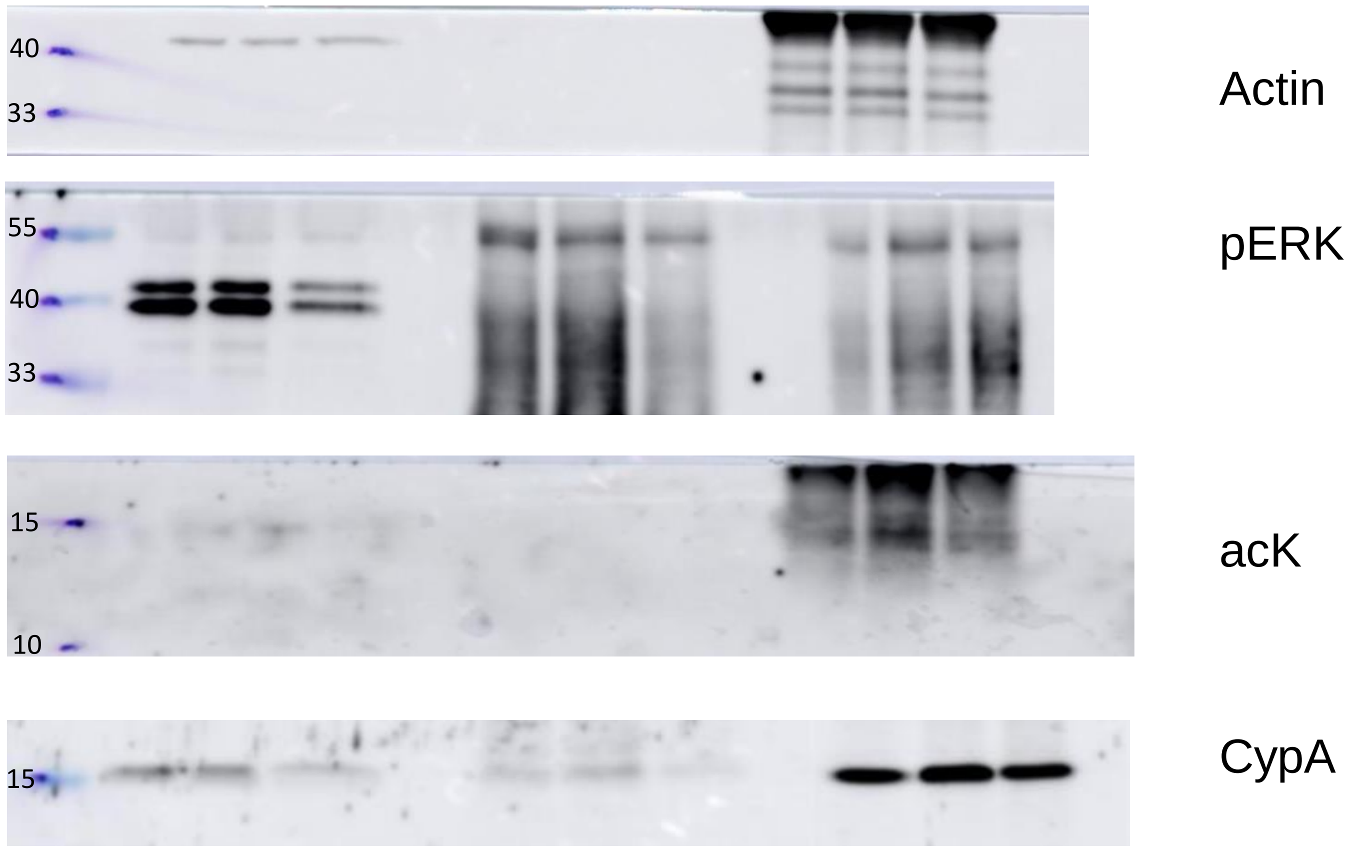

Supplement: Supplementary file 1 [file ijms-23-01469-s001.zip › ijms-1545228-Uncropped Western Blot images.pdf]
